# The use of integrin binding domain loaded hydrogel (RGD) with minimally invasive surgical technique in treatment of periodontal intrabony defect: a randomized clinical and biochemical study

**DOI:** 10.1590/1678-7757-2023-0263

**Published:** 2023-12-22

**Authors:** Shaimaa Hamdy ABD EL-AZEEM, Ahmed Abdallah KHALIL, Mohammed Abdel-Moniem IBRAHIM, Ahmed Y GAMAL

**Affiliations:** 1 Nahda University Faculty of Dentistry Oral Medicine Oral Diagnosis and Periodontology Department Beni Swef Egypt Nahda University, Faculty of Dentistry Oral Medicine, Oral Diagnosis and Periodontology Department, Beni Swef, Egypt.; 2 Minia University Faculty of Dentistry Oral Medicine Oral Diagnosis and Periodontology Department Minia Egypt Minia University, Faculty of Dentistry Oral Medicine, Oral Diagnosis and Periodontology Department, Minia, Egypt.; 3 Ain Shams Universit Faculty of Dentistry Oral Medicine Oral Diagnosis and Periodontology Department Cairo Egypt Ain Shams Universit, Faculty of Dentistry Oral Medicine, Oral Diagnosis and Periodontology Department, Cairo, Egypt.; 4 Misr University for Science and Technology Faculty of Dentistry Cairo Egypt Misr University for Science and Technology, Faculty of Dentistry, Cairo, Egypt.

**Keywords:** Periodontal regeneration, Minimally invasive surgical technique, RGD peptide, Hydrogel, Integrin

## Abstract

Periodontal regeneration faces multiple challenges, the most important being cellular insufficiency. In an attempt to improve defect cellularity, we aimed to demonstrate enhancing cellular attraction using arginine-glycine-aspartic acid (RGD) adhesion molecule legend blended hydrogel within the intrabony defects.

**Methodology:**

Forty-five intrabony defects were selected from patients with stage III or IV - grade A or B periodontitis and divided randomly into three equal groups of 15 each: group1 (G1): received minimally invasive surgical technique (MIST) alone, group2 (G2): received MIST and placebo hydrogel injection, and group3 (G3): were treated with MIST and RGD hydrogel injection. Primary outcomes 6 months following therapy were; defect base fill (DBF) and defect width measurement (DW); secondary outcomes were clinical attachment level (CAL), pocket depth (PD), plaque index (PI), gingival index (GI), and biochemical analysis of bone morphogenetic protein (BMP-2) evaluated at 1,7,14 and 21 days following therapy.

**Results:**

Significant improvements in DBF, CAL, and PD were observed in the three studied groups 6 months following therapy compared to baseline (p<0.05). A significant improvement in DBF was reported in G3 compared to G1 and 2 (p=0.005). Additionally, a significantly higher CAL gain was reported in G3 compared to that of G1 (p=0.02). Group 3 was associated with a significantly higher level of BMP-2 compared to G1 and G2 in all reported periods.

**Conclusion:**

RGD peptide carried on a hydrogel delivery agent and contained with a minimally invasive flap could be a reliable option in improving the outcomes of periodontal therapy.

## Introduction

Periodontal regeneration is a complex process that involves coordinated activities and interactions of many cell types, extracellular matrix, cytokines, and specific growth factors to restore tissue integrity. The most important challenge facing periodontal regeneration is cellular insufficiency.^1^ Periodontal defects usually have a limited regenerative capacity due to their bounded surface area that is supposed to provide the wound area with a limited number of viable cells and a limited number of biologic mediators.^2^ Cell recruitment and adhesion into the defect area are essential for cells to survive and secrete collagen.^3^

Apoptosis is initiated when failure in adhesion in many different cell types occurs.^4^ Some periodontal treatment options failed in the reconstruction of the defect due to failure in wound stabilization and subsequent cell adhesion. Many treatment options have been developed to enhance defect stability and cellular recruitment including the use of GTM and different biologics. However, the treatment outcomes vary considerably depending on the level of the defect cellularity and the degree of cell recruitment into the defect area.^5^ For maximum outcomes, enhanced stability, vascularity, and biologics-sustained delivery have been suggested.^6^ Among them, there is the minimally invasive surgical technique (MIST) suggested by Cortellini and Tonetti^7^ (2007), which offers a suitable level of tissue preservation that could help in defect stability and cellular adhesion. It has been suggested to promote flap stability, maintain space**,** and maintain a greater amount of blood supply at the alveolar crest and papillary levels.^3^ MIST could be an effective option to be used with biologics and its delivery vehicles for more enhanced containment.^4^ To enhance cellular attraction and adhesion, the most widely used adhesion ligand, which is known to affect how cells respond, is cationic charges tri-amino acid sequences known as RGD.^8^ RGD peptide is the integrin binding site in many ECM proteins such as fibronectin, osteopontin, vitronectin, and bone sialoprotein that allow cell adhesion and differentiation.^9^ RGD peptide was reported to improve periodontal ligament cells (PDLCs) adhesion and induced osteogenic proliferation and differentiation of these cells.^4^

Hydrogels are natural or synthetic biocompatible polymers that are widely used in periodontal tissue engineering as a delivery material for different biologics.^10^ Synthetic hydrogels show superiority in chemical and mechanical properties over natural hydrogels.^9^ Carbopol, or polyacrylic acid (PAA), is one of the most widely used polymers as hydrogel. It is an anionic polyelectrolyte that can be readily polymerized and crosslinked to form hydrogels that have a swelling capacity that is greater than their dry weight.^11^ Numerous studies have been performed on the usage of Carbopol for biomedical applications due to both its high swelling capabilities and

the convenience with which cationic molecules can be loaded for its polyanionic charge.^12^^,^^13^ Carbopol hydrogel can also significantly improve the stability of its loaded biological mediators.^14^

Despite its proven potential in enhancing cellular adhesion, RGD significance in periodontal regeneration is insufficiently studied. To maximize cellular recruitment and improve defect stability, this study was conducted to clinically and biochemically evaluate the effect of using RGD peptide carried on hydrogel in treating human intrabony defect using the MIST approach.

## Methodology

### Population

This randomized double-blinded controlled clinical trial included 45 patients, each contributing with one periodontal intrabony defect. Patients were selected from the Medicine, Oral Diagnosis, & Periodontology Department of the Faculty of Dentistry, Minia University outpatient clinic from May 2020 to December 2021. The International Conference on Harmonization Good Clinical Practice Guideline, the Declaration of Helsinki, and the research ethics committee guidelines of the Faculty of Dentistry, Minia University were all followed during the study (No. 466 November 30, 2020). This study was registered in ClinicalTrials.gov U.S. National Library of Medicine under the number NCT05653245.

### Inclusion and exclusion criteria

Patients were included based on the following criteria: 1- Medically free, from both sexes, and in the age range of 20-55 years old. 2- Stage III or IV, grade A or B periodontitis.^15^ 3- Contained 2- or 3-wall osseous defect detected by CBCT. 4- Vital teeth or teeth treated duly with root canal treatment. 5- Optimal compliance during the cause-related phase of therapy. 6- No antibiotics or medication use during the last six months. Exclusion criteria were; 1- Smokers, 2- Pregnant or lactating females, 3- Patients with full-mouth plaque score ≥ 20% and full-mouth bleeding score ≥ 20% following Phase I therapy, 4- Mobile teeth and third molars. All patients signed an informed consent form on the study’s aims and all risks and advantages of the therapeutic procedures were disclosed.

### Randomization, allocation, and blinding

**Figure 1:**
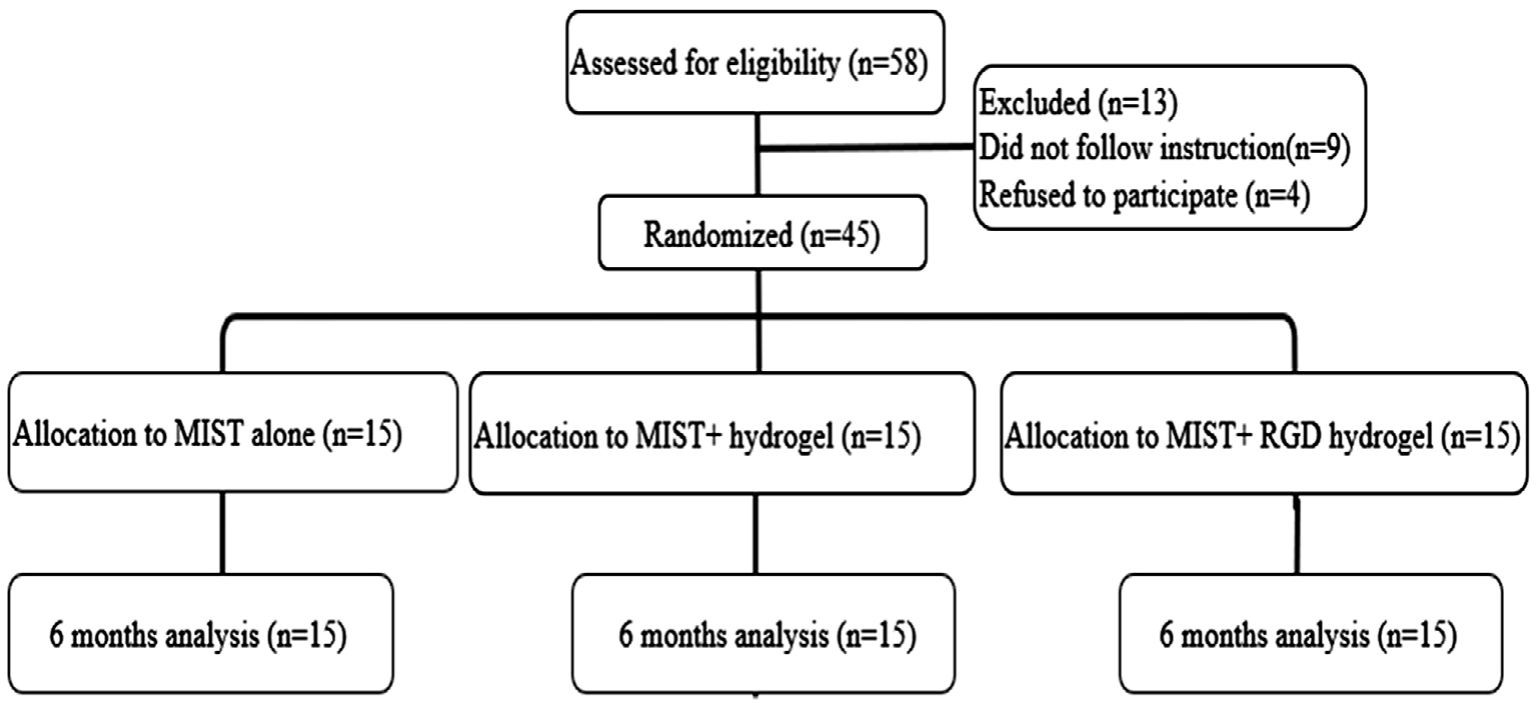
Consort diagram for the study

Participants were randomly assigned using a computerized random number generator (Available from: www.random.org) into three parallel groups, with 15 participants each. Group 1: intrabony defect sites underwent MIST alone. Group 2: underwent MIST and placebo hydrogel injections. Group 3: underwent MIST and RGD hydrogel injection (Figure 1). The allocation sequence was produced by software and preserved in invisibly sealed envelopes. Clinical periodontal parameters were recorded by the same blinded evaluators (A.G). The evaluator, operator (Sh. H), and all patients were blinded regarding the hydrogel-loaded or unloaded treatment option.

### Sample size calculation

Power analysis was designed to have appropriate power to apply a statistical test of the null hypothesis, which implied that no difference would be found in bone healing between different groups. By adopting an alpha level of (0.05) a beta of (0.2), i.e., power=80% and an effect size (f) of (0.479), calculated based on the results of a prior study;^16^ the predicted sample size (n) consisted of (45) samples (15 samples per group). Sample size calculation was performed using G*Power version 3.1.9.7.^17^

### Interventions

#### Hydrogel preparation

A total of 50 mg of Carbopol® 940 (Lubrizol Corporation, US) was impregnated in 10 ml water, remaining under a magnetic stirrer until completely dissolved. pH and viscosity were adjusted with triethanolamine (TEA) until the required viscosity was approximately reached (40,000–60,000 mPa-s).^18^ The stability of prepared hydrogel was reported for four weeks.^13^

#### Preparation of 2.5 mM RGD *Hydrogel*19

A total of 10 mg of RGD^®^ peptide (Sigma Aldrich, A-8052, Darmstadt, Germany) was soaked in 11.5 ml distilled water, remaining under a magnetic stirrer after being dissolved. Then, 50 mg of Carbopol® was added and stirred. TEA drops were added until the needed viscosity was approximately reached (40,000–60,000 mPa-s).^18^

#### Pre-surgical preparation

Supra and subgingival instrumentation were performed using a universal curette and ultrasonic instrument (woodpecker U600, KMC). Pre-surgical preparation was performed following the EFP S3 guidelines.20 Step 1: full mouth supragingival scaling in one visit together with patient education and oral hygiene instructions. Step 2: therapy following clinical improvement of gingival

inflammation and subgingival instrumentation was performed in one visit. Adjunctive chemical plaque control of chlorhexidine digluconate (Antiseptol, Cairo Pharmaceuticals, and Chemical Industries Co., Egypt) mouth rinse 0.12% twice daily was prescribed. One month following Step 2 therapy, patients with a full-mouth plaque score of ≤ 20%, a full-mouth bleeding score of ≤ 20%, and a score of 0 at the selected site were included in the study.^21^ Initial clinical data, photographs, and radiographs were documented.

#### Outcome measures

Clinical and radiographic parameters were obtained four weeks after (S2) Phase 1 therapy was completed (baseline) and six months after surgery. The primary outcome was the evaluation of clinical attachment level (CAL), defect base fill (DBF), crestal bone level (CBL), and defect width (DW). The secondary outcomes were to evaluate the soft tissue parameters and BMP-2 level.

**Figure 2:**
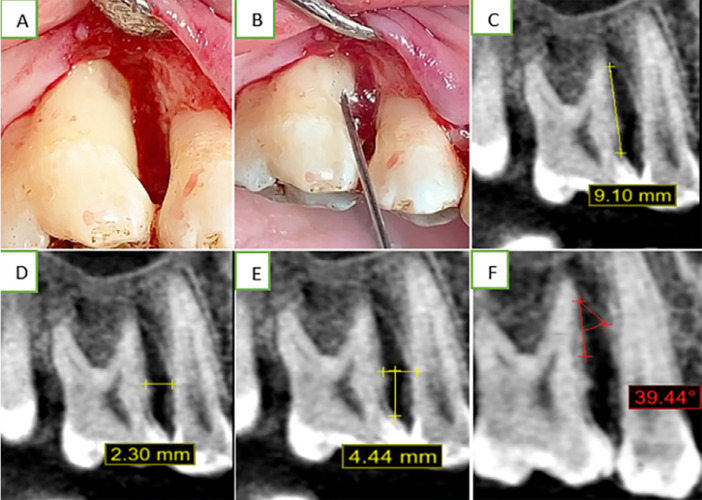
(A) MIST for 2-wall intrabony defect incision; (B) RGD loaded hydrogel application; (C) baseline radiographic measures of CEJ-BD= 9.10 mm; (D) radiographic width of bone defect= 2.3 mm; (E) radiographic measures of CEJ-BC= 4.44 mm; (F) defect angle= 39.44°

Radiographic evaluation: Quantitative radiometric and geometric analysis of alveolar bone changes was performed pre-operatively and six months following treatment using cone beam computed tomography (CBCT). For each defect, three measurements were taken: a- DBF, the linear distance from the cement-enamel junction (CEJ) to the base of intrabony defect (CEJ-BD) (Figure 2C) b- CBL, the linear distance from CEJ to the bone crest of the involved tooth (CEJ-BC) (Figure 2E), and c- DW, the angle between the root surface and bony wall of the pocket (Figure 2D).^22^ The method for computer-assisted CEJ detection in intraoral ultrasounds entails image preprocessing procedures for image enhancement, segmentation, and edge detection to identify the border of the enamel.

Probing Depth (PD): measurements were recorded by UNC^15^ periodontal probe from the free gingival margin to the deepest interproximal part of the defect.^23^ Clinical attachment level (CAL): was measured from the CEJ to the bottom of the pocket. Plaque Index (PI)^24^ and Gingival Index (GI)^25^ were also reported at baseline and six months following surgery. All clinical parameters were measured from the same interproximal point using a customized acrylic stent.

Biochemical evaluation: Gingival Crevicular Fluid (GCF) samples were collected by paper point size 30 (META BIOMED^®^ Co., Ltd- Korea & international) inserted 2 mm into the pocket, kept in position for 30 seconds.^26^ The collected GCF was immediately transferred into an Eppendorf tube containing phosphate buffer saline (PBS; 137 mm NaCl, 10 mm Na2HPO4, and 2.7 mm KCl; pH 7.3). Samples were frozen at -80°C until they were assayed for BMP-2 levels using enzyme-linked immunosorbent assay ELISA^®^ (Elisa, ELK Biotechnology CO., LTD. China). GCF samples were collected on days 1, 7, 14, and 21 following surgery.

#### Surgical intervention

Selected sites were anesthetized using articaine HCl 4% with adrenaline (1:100,000) (Artinibsa, inibsaSpain, wedjat pharma). A minimally invasive surgical flap was performed as described by Cortellini and Tonetti^7^ (2007). Briefly, the interdental papilla was surgically approached by MPPT or SPPT, depending on whether interdental space was ≥ 2 mm or ≤ 2 mm, respectively. A sharp intrasulcular incision using No#67 fine micro-blades with the aid of magnifying loupe was performed to preserve all gingival height and width. The buccal and lingual full-thickness flaps with minimum mesiodistal and coronal-apical extensions were performed to expose the coronal margin of the remnant bone crest. The buccal and lingual intrasulcular incisions were limited to the teeth adjacent to the defect, and flaps were elevated with tiny periosteal elevators to uncover the defect and the residual bone crest (Figure 2A).^27^ Complete debridement was performed by sharp mini-curettes (Gracey, Hu-Friedy). RGD hydrogel was applied to overfill the defect using a plastic syringe in Group 3 (Figure 2B), whereas hydrogel alone was used in Group 2 defects. All surgeries were performed by the same experienced periodontist (Sh. H). A single modified internal mattress suture was conducted to get a tension-free primary closure. (6-0 polypropylene monofilament industrial zone, Borg El Arab El Gedida city-Alex. Egypt). Patients received post-surgical instructions that included avoiding mechanical plaque control at the surgical site and using 0.12% chlorhexidine mouthwash three times per day for one week.^28^ Ibuprofen 600 mm was used twice daily for three days to manage discomfort following surgery, along with systemic doxycycline (100mg b.i.d. for one week) to control infection.

#### Data analysis

Numerical data were presented as mean and standard deviation values. They were tested for normality using Shapiro-Wilk’s test. PI and GI were non-parametric and were analyzed using Kruskal-Wallis test. Radiographic changes in bone defect (CEJ-BD, crestal bone level, and defect width) were statistically analyzed by paired t-test for intragroup comparisons and by covariance (ANCOVA) to compare the mean changes between the three groups using baseline data as a covariate. BMP-2 statistical analysis was performed by repeated measure ANOVA for data collected after 1 day, 7 days, and 14 days. P value of <0.05 was considered statistically significant.

## Results

### Study population

In this double-blinded randomized clinical trial, 45 patients were enrolled and divided into three groups, all patients bone loss/ age were calculated to be ≤ 1. Group 1, MIST alone, was performed for 15 sites (8 males and 7 females with a mean age of 34.80±7.07); Group 2, MIST + placebo hydrogel applied in 15 sites (5 males and 10 females with a mean age of 34.50±6.31); and Group 3, MIST + RGD loaded hydrogel defect overfill for 15 sites (6 males and 9 females with a mean age of 34.70±6.88). No participant dropped out during the study period, and no data points were missing from the analysis (Table 1).

**Table 1:** Demographic characteristics

	Group1	Group2	Group3	p/ value
Gender (m/f)	8/7	5/10	6/9	
Mean age (years)	34.80±7.07	34.50±6.31	34.70±6.88	1.00

### Radiographic outcomes (primary outcomes)

**Figure 3:**
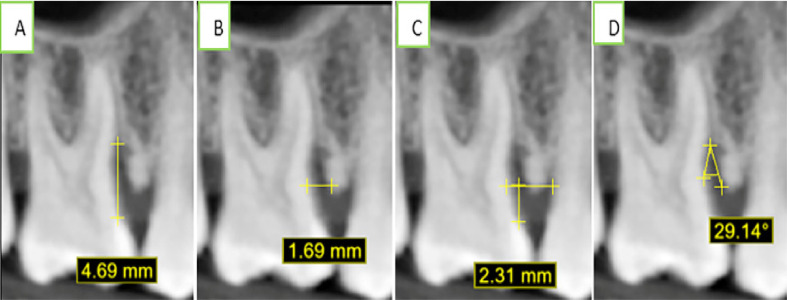
Radiographic measures after 6 months follow up. (A) defect bone fill after 6 months= 4.69 mm; (B) defect width= 1.69 mm; (C) crestal bone formation= 2.31 mm; (D) angle measure= 29.14°

Significant differences between baseline and 6-month measurements of DBF were observed in all groups (Figure 3). In Group 1, mean distance was reduced from 9.83±1.00 to 5.01±0.89; in Group 2, from 9.64±1.23 to 4.86±0.79; and, in Group 3, from 10.25±0.74 to 3.98±0.52 (p value= 0.00*). Defect base fill was significantly higher in G3 compared to G1 (p value= 0.005*) and G2 (p value= 0.014*), with a 1.137 and 1.029 mean difference between G1 and G3 and between G2 and G3, respectively. CBL at baseline and 6 months demonstrated a significant difference in all groups. In Group 1, the CBL was reduced from 3.76± 1.13 to 3.39± 1.16 (p= 0 .008*); Group 2 from 3.53± 1.22 to 3.07± 1.18 (p= 0.003*); and Group3 from 3.85± 1.27 to 2.71±0.85 (p value= 0.001*). A statistically significant higher reduction was observed in G3 compared to G1 negative control after mean adjustment. A mean difference between the two groups of 0.76 with p= 0.003* was reported, along with a significant difference between G3 and G2 p= 0.018* and in the mean difference between the two groups, which was 0.62. Comparison between baseline and 6 months bone width reported that all three groups presented a significant reduction. Group 1 mean defect width reduced from 3.24±0.56 to 2.63±0.44 (p value= 0.002*); Group 2 reduced from 3.32±0.52 to 2.64±0.40 (p= 0.006*); and Group 3 reduced from 3.48± 0.60 to 2.37± 0.68 (p= 0.006*). No significant differences were reported between the three groups after mean adjustment by ANCOVA test as p> 0.05.

### Plaque index and gingival index

After six months, no significant differences were reported for GI and PI scores. The PI mean was reported to be (0.4±0.7) in Group 1 and (0.4±0.52 and 0.4±0.52) in Groups 2 and 3, respectively. There was no statistically significant difference between baseline and 6-month follow-up between the three groups (p > 0.05) and no significant difference between the three groups (P > 0.05). Again, no significant differences were reported at 6-month GI compared to baseline. Mean GI was 0.60±0.52 at baseline and 0.7±0.67 after 6 months in Group 1, and changed from 0.60±0.70 to 0.50±0.52 in Group 3, remaining constant in Group 2 (P > 0.05); thus, no statistically significant difference was noted between the three groups (P > 0.05) (Table 2).

**Table 2:** Mean PI and GI data at baseline and 6 month evaluation periods

Parameters		Group 1	Group 2	Group 3	P value
		mean±S.D	mean±S.D	mean±S.D	
PI	Baseline	0.50±0.70	0.30±0.48	0.50±0.53	0.68
	6months	0.40±0.70	0.40±0.52	0.40±0.52	0.95
	P/value	0.66	0.56	0.56	
GI	Baseline	0.60±0.52	0.50±0.53	0.60±0.70	0.92
	6 months	0.7±0.67	0.50±0.53	0.50±0.52	0.76
	P/value	0.66	1	0.66	

### Clinical attachment Level and pocket depth outcomes

Clinical attachment level and pocket depth showed a significant reduction from baseline to 6-month measurements in the three studied groups (p value= 0.000*). In G1 mean CAL reduced from 8.9±0.99 to 4.3±0.95, group 2 from 8.8±1.48 to 4.1±0.74 and in G3 from 9.5±1.08 to 3.5±0.53. A statistically significant higher reduction was observed in G3 compared to G1 (p= 0.020*) with a mean difference of 0.954 between the two groups. In G1 mean pocket depth reduced significantly from 7.50±1.26 to 3.90±0.88, G2 reduced from 7.60±1.34 to 3.90±0.74 and G3 from 8.60±1.34 to 3.20±0.42. No significant difference between groups after mean adjustment as p>0.05 (Table 3).

**Table 3:** Changes in clinical and radiographic parameters over time and comparisons between groups

Parameter	Group	Baseline	6 months	Mean difference in mm	P value	Partial eta squared (η2)	Comparison between groups	P value between groups
		mean±S.D	mean±S.D					
CAL	Group 1	8.90±0.99	4.30±0.95	4.6	0.000*	0.27	Group 1 Group 2	1
	Group 2	8.80±1.48	4.10±0.74	4.7	0.000*		Group 1 Group 3	0.02*
	Group 3	9.50±1.08	3.50±0.53	6	0.000*		Group 2 Group 3	0.07
PD	Group 1	7.50±1.26	3.90±0.88	3.6	0.000*	0.2	Group 1 Group 2	1
	Group 2	7.60±1.34	3.90±0.74	3.7	0.000*		Group 1 Group 3	0.096
	Group 3	8.60±1.34	3.20±0.42	5.4	0.000*		Group 2 Group 3	0.095
CEJ-BD (DBF)	Group 1	9.83± 1.00	5.01±0.89	4.82	0.000*	0.36	Group 1 Group 2	1
	Group 2	9.64± 1.23	4.86±0.79	4.78	0.000*		Group 1 Group 3	0.005*
	Group 3	10.25±0.74	3.98±0.52	6.27	0.000*		Group 2 Group 3	0.014*
Defect width (DW)	Group 1	3.24±0.56	2.63±0.44	0.61	0.002*	0.07	Group 1 Group 2	1
	Group 2	3.32±0.52	2.64±0.40	0.68	0.006*		Group 1 Group 3	0.76
	Group 3	3.48± 0.60	2.37± 0.68	1.11	0.006*		Group 2 Group 3	0.73
CEJ-BC (CBL)	Group 1	3.76± 1.13	3.39± 1.16	0.37	0 .008*	0.37	Group 1 Group 2	1
	Group 2	3.53± 1.22	3.07± 1.18	0.46	0.003*		Group 1 Group 3	0.003*
	Group 3	3.85± 1.27	2.71±0.85	1.14	0.001*		Group 2 Group 3	0.02*

### Biochemical outcomes of BMP-2 release profile

Gingival crevicular fluid BMP-2 levels showed a significant increase in its level throughout the selected evaluation periods in all groups. In G1, there was an increase from a mean of 223.85±7.27 on day 1, to 307.64±16.98 on day 7 and 333.15±31.62 on day 14, reaching the optimum level on day 21 with a mean level of 358.25±40.93. In G2, mean value on day 1 was 227.56±12.08; on day 7, 297.53±45.00; on day 14, 335.00±38.73; and on day 21, 368.00±30.54. In G3, mean level on day 1 was 225.70±7.813; on day 7, 361.94±21.49; on day 14, 385.35±10.17; and increased to 434.00±19.92 on day 21. A significantly higher level of BMP-2 was reported in G3 compared to G1 and G2 (p= 0.036 and p= 0.041), respectively. No significant difference between G1 and G2 was reported (p= 0.996). (Table 4).

**Table 4:** Changes in BMP-2 - intragroup comparisons and comparisons between groups over time

	Group1	Group2	Group3			
Time	(Mean±SD)	(Mean±SD)	(Mean±SD)	Compare between groups		p/value
Day 1	223.85±7.27	227.56±12.08	225.70±7.813	Group 1	Group 2	0,996
Day 7	307.64±16.98	297.53±45.00	361.94±21.49		Group 3	0.036*
Day 14	333.15±31.62	335.00±38.73	385.35±10.17	Group 2	Group 3	0.041*
Day 21	358.25±40.93	368.00±30.54	434.00±19.92			
p/value	0.000*	0.000*	0.000*			

## Discussion

The main three elements required for optimal regeneration are defect stability, space maintenance, and enhanced cellular and mediators’ availability. The present study suggested a combination of MIST—supposed to provide reliable defect stability, space maintenance, and growth factors containment—with RGD adhesion protein hydrogel, which was hypothesized to improve cellular availability since both options are capable of providing optimal outcomes. MIST was used instead of papilla preservation technique due to possible papilla issues, such as collapse or complete necrosis that lead to biomaterial exposure.^29^ Hydrogels are hydrophilic structure, which provides physical properties close to natural tissue.^30^ Carbopol presents a good swelling capacity in a neutral state and is easily crosslinked in hydrogels. For tissue regeneration, it is used as a drug carrier with increased muco-adhesiveness, which increases the drug bioavailability^31^ since an essential need for the long-term survival of migrated cells is cell attachment.^32^

Cell adhesion peptide RGD was reported to be incorporated into biomaterials to improve cell adherence due to the bioinert nature of most synthetic materials.^9^ Moreover, only Intrabony supportive defects were included to insure maximum RGD containment within the defect for proper evaluation of its clinical impact. RGD adhesive peptide proved effective in stimulating the attachment of many different cell types to various kinds of scaffolds.^33^ In this study, RGD peptide concentration was 2.5 mM since it is the maximum reported concentration used for bone markers protein production.^19^ Moreover, BMP-2 levels were evaluated within the GCF since it is considered an important mediator for bone formation. BMP-2 promotes the transformation of PDL cells into osteoblasts and increases the expression of markers for mineralized tissue.^6^ BMP-2 stimulated ALP activity, promoted mineralization, improved adhesion, and mediated the production and activation of specific related osteogenic markers, which helped bone mesenchymal stem cells differentiate into osteoblasts.^34^ GCF sample was collected a day after surgery since samples taken the day of surgery were frequently tainted with blood and, in the following day, it was better to capture the acute effects of the surgical intervention on BMP-2 levels in the early stages of healing.

A statistically significant improvement was observed in bone fill measured from CEJ to BD and from CEJ to BC in all groups, which may be explained by the effect of MIST space-making and enhanced blood clot stability that were reported in many studies.^2^^,^^35^^,^^36^ G3 shows a more significant bone fill level compared to G1 and 2. This could be attributed to RGD peptide enhanced cell adhesion effect and hydrogel capacity to retain natural BMP-2 within the defect area. Lin, et al.^37^ (2019) and Alipour, et al.^38^ (2020) reported that RGD-modified titanium alloy improved osteoblasts early adhesion and distribution, as well as later proliferation and differentiation. RGD peptide in a dose of 2.5 mM was reported to increase alkaline phosphatase (ALP) production, which is considered the early bone marker crucial for ossification.^19^

In this study, clinical attachment gain and pocket reduction were reported to be improved at 6-month evaluation period in all groups when compared to baseline. These findings highlight the significance of minimally invasive approaches in improving soft tissue parameters. Many studies claimed that MIST improved soft tissue stability, which is supposed to maintain and increase blood supply to the involved papilla and alveolar crest area.^39^ A statistically significant difference between G1 and G3 was observed as RGD peptide can stimulate the migration and proliferation of fibroblasts, which play a key role in tissue repair. RGD also was reported to promote angiogenesis.^40^ G1 and G2 they were used as a separate group to exclude the effect of MIST alone and effect of placebo hydrogel when used alone (without RGD).

GCF analysis of BMP-2 reported a significant increase in its levels between days 1, 7, 14, and 21 (p=0.000) in the three groups. This could be also explained by the capacity of MIST to retain growth factors within the defect area without being rabidly washed away by crevicular fluid flow. The level of BMP-2 was reported significantly higher in Group 3 compared to Groups 1 and 2. This significant value may be explained by the presence of RGD peptide, which is supposed to stimulate cell migration, adhesion, and growth factors production, including BMP-2. Again, hydrogel could improve secreted growth factor availability and maintain its high level throughout the studied intervals. The addition of adhesive RGD peptide was reported to increase the osteoinductive impact of the BMP-2 peptide twofold.^34^

## Conclusion

Within the limitations of this study, which, to the best of our knowledge, is the first study that evaluated the clinical and biochemical significance of RGD hydrogel in periodontal regeneration, we can conclude that the use of RGD peptide-loaded hydrogel could be an effective treatment option that improved periodontal regeneration. MIST could be important in maintaining RGD peptides and autogenous growth factors levels within the defect area for more positive outcomes. More studies are required to evaluate the minimum amount of RGD molecules required to get the maximum effect on cellular adhesion.
